# ALDH3B1 protects interfollicular epidermal cells against lipid peroxidation via the NRF2 pathway

**DOI:** 10.1007/s12192-022-01306-9

**Published:** 2022-11-03

**Authors:** Zhenjie Wu, Aoyu Chen, Guang Zhang, Chunyan Liu, Siyuan Yin, Ru Song, Jiaxu Ma, Guoqi Cao, Rui Sun, Jian Liu, Yibing Wang

**Affiliations:** 1 Department of Plastic Surgery, Shandong Provincial Qianfoshan Hospital, Shandong University, Jinan, Shandong 250012 People’s Republic of China; 2 Department of Plastic Surgery, The First Affiliated Hospital of Shandong First Medical University & Shandong Provincial Qianfoshan Hospital, Jinan, Shandong 250014 People’s Republic of China; 3 Jinan Clinical Research Center for Tissue Engineering Skin Regeneration and Wound Repair, The First Affiliated Hospital of Shandong First Medical University & Shandong Provincial Qianfoshan Hospital, Jinan, Shandong 250014 People’s Republic of China

**Keywords:** Holotransferrin, ALDH3B1, Interfollicular epidermal (IFE) cells, Lipid peroxidation, NRF2

## Abstract

Reactive oxygen species (ROS) production is critical for the initiation of wound repair; however, persistently high levels of ROS can lead to lipid peroxidation in cells and thus affect wound healing. Iron is a transition metal that is an essential component of almost all living cells and organisms. When present in excess in cells and tissues, iron disrupts redox homeostasis and catalyzes the generation of ROS, leading to increased lipid peroxidation. In this study, we found that after treating interfollicular epidermal (IFE) cells with different concentrations of holotransferrin (0 µg/ml, 1 µg/ml, 10 µg/ml, 100 µg/ml, and 1 mg/ml), the intracellular iron content increased, and cell viability and function did not differ significantly among the treatment groups of cells. In addition, the level of lipid peroxidation in IFE cells did not increase following holotransferrin treatment. We speculated that there is a protective mechanism within IFE cells that reduces the occurrence of intracellular lipid peroxidation. We also found that the elevated intracellular iron content of IFE cells was accompanied by elevated ALDH3B1 expression. We investigated the effect of ALDH3B1 on the level of lipid peroxidation in IFE cells and found that elevated ALDH3B1 expression decreased the damage to IFE cells induced by lipid peroxidation. In addition, the NRF2 pathway was found to affect the expression of ALDH3B1, which in turn affected lipid peroxidation in IFE cells. These findings suggest that in IFE cells, activation of the NRF2 pathway can increase the expression of ALDH3B1 and thus reduce the production of intracellular ROS and the occurrence of intracellular lipid peroxidation. Therefore, ALDH3B1 may be a potential target for the treatment of chronic wounds.

## Introduction

The failure of wounds to heal causes great pain to patients and places an economic burden on society (Zuk et al. 2001). Wound healing is an important and complex physiological process that is essential to maintain the barrier function of the skin and consists of a haemostatic/inflammatory phase, a proliferative phase, and a remodelling phase (Gosain and DiPietro 2004; Guo and Dipietro 2010; Martin 1997; Singer and Clark 1999). During the progression of many diseases, events related to wound healing can be compromised, leading to the nonhealing of the wound and the formation of chronic wounds (Brem et al. 2007; Schultz et al. 1987). Reactive oxygen species (ROS) play a key role in the orchestration of the normal wound healing response, and ROS-mediated redox signalling is involved in various processes, such as cellular recruitment and cytokine and growth factor production (Cano Sanchez et al. 2018). ROS are essential mediators of intracellular signals for hemostasis, vascular regeneration, and re-epithelialization and are crucial for stimulating effective wound healing (D’Autréaux and Toledano 2007). However, the excessive release of ROS leads to impaired repair of cellular damage and wounds, lipid peroxidation, and cell death through apoptosis, which is the main cause of chronic wounds (Schäfer and Werner 2008). Prolonged nonhealing of chronic wounds can lead to the extravasation of red blood cells, resulting in an iron deposition. Loading of iron by macrophages (via erythrophagocytosis) leads to unrestricted macrophage promotion of inflammatory activation. The generated ROS cause a cascade of deleterious reactions that increase lipid peroxidation. Holotransferrin has been found to increase intracellular iron levels, resulting in an increase in the cellular production of ROS and thus an increase in intracellular lipid peroxidation levels. We chose the holotransferrin experimental model to simulate the environment of increased iron content in chronic trauma and to study the changes after increases in intracellular iron content in interfollicular epidermal (IFE) cells (Wright et al. 2014).

Iron is an important factor in the maintenance of healthy skin, and the skin is one of the main organs of iron metabolism; iron is actively excreted from the body through skin desquamation (Wright et al. 2014). When iron is ingested by the body, it is absorbed as Fe^2+^ and then bound to transferrin in the form of Fe^3+^, which is imported into cells via the membrane protein transferrin receptor 1 (TfR1). After entering a cell, Fe^3+^ is reduced to Fe^2+^ by reductase. Under normal physiological conditions, redox-active ferrous ions are maintained at low concentrations in the form of unstable iron pools to sustain metabolic requirements, and excess ferrous ions are sequestered in proteins involving ferritin to avoid toxic reactions (Feng et al. 2022). Iron is a powerful catalyst of lipid peroxidation, and high intracellular iron concentrations generate large amounts of ROS in the Fenton reaction, leading to elevated levels of lipid peroxidation; this process leads to cell death and has important implications for chronic wound formation (Wright et al. 2014; Sindrilaru et al. 2011). Although the underlying mechanisms have not been thoroughly investigated, it is agreed that iron affects wound healing by regulating lipid peroxidation (Wlaschek et al. 2019).

Keratin-forming cells derived from interfollicular epidermis and hair follicles play different but important roles in wound healing; as the epidermis remodels after wound healing, keratin-forming cells of these different origins lose their original characteristics and eventually assume an interfollicular epidermis-like phenotype (Abhishek and Palamadai 2016; Clayton et al. 2007; Gonzales and Fuchs 2017; Mardaryev et al. 2014), becoming important in tissue repair. Therefore, it is crucial to identify the mechanisms that contribute to the resistance of IFE cells to lipid peroxidation.

ALDH3B1 is an aldehyde dehydrogenase that plays an important role in the detoxification of cellular aldehydes (Marchitti et al. 2010). Therefore, we hypothesized that ALDH3B1 plays a role in the resistance of IFE cells to lipid peroxidation. We found that under H_2_O_2_ treatment conditions, the ability of cells to resist lipid peroxidation gradually increased as the intracellular expression of ALDH3B1 increased. The NRF2 pathway plays an important role in cellular resistance to oxidative stress; the activation of this pathway protects cells from oxidative stress damage and increases cell survival (Li et al. 2018). Activation of NRF2 under pathological conditions has antioxidant properties, and NRF2 controls inflammation by inhibiting the production of ROS and the expression of inflammatory cytokines, which improves angiogenesis and promotes wound healing (Li et al. 2018; Lu et al. 2016; Ma et al. 2019). In addition, NRF2 can bind to the ALDH3B1 promoter and regulate the expression of ALDH3B1 (Namani et al. 2017). Therefore, we speculated that IFE cells exert resistance to lipid peroxidation through the NRF2 pathway, and we experimentally demonstrated that IFE cells protect themselves from lipid peroxidation damage by elevating ALDH3B1 expression through the NRF2 pathway.

## Material and methods

### Animals

A total of 30 specific pathogen-free (SPF) wild-type male C57BL/6 J neonatal mice (license: SCXK Lu20190001) were purchased from Shandong University Laboratory Animal Center. All experimental animals were housed at 22 ± 1 °C under 50 ± 1% relative humidity and a 12/12-h light/dark cycle.

### Cell isolation, culture, processing, and transfection

In this research, newborn mice for primary cell extraction were purchased from Shandong University Laboratory. All experiments were approved by the ethics committee of The First Affiliated Hospital of Shandong First Medical University & Shandong Provincial Qianfoshan Hospital (approval number: SYDWLS (2021)002) and were performed in accordance with the guidelines and regulations. The isolation and culture of cells were performed according to previous studies (Clayton et al. 2007; Gonzales and Fuchs 2017; Aliborzi et al. 2016; Wang et al. 2022; Singh 2022; Ghadially 2012). However, we extended the adhesion time to 1 h to obtain IFE cells. IFE cells were cultured in holotransferrin (Germany, Sigma) medium (Cellntec, Switzerland) containing different concentrations of holotransferrin (Germany, Sigma; 0 µg/ml, 1 µg/ml, 10 µg/ml, 100 µg/ml, and 1 mg/ml) for 48 h. All cells were cultured at 37 °C with 5% CO2. For ALDH3B1 overexpression experiments, IFE cells were infected with Flag-ALDH3B1 adenovirus and Flag-control adenovirus (GeneChem Co., Ltd., Shanghai, China) for 10 h. The multiplicity of infection (MOI) was determined to be 30. IFE cells were transfected with 50 nM of small interfering NRF2 (siNRF2; Guangzhou RiboBio Co., Ltd., Guangzhou, China) or a negative control (siNC; RiboBio) using RiboBio transfection reagent. Forty-eight hours later, the cells were harvested for RNA and protein extraction and assay. Cells were incubated with the iron death inducer RSL3 (MedChemExpress, 5 µM) and erastin (MedChemExpress, 20 µM) for 6 h. The sequence of siNRF2 small interferences used is shown below.

si-m-Nfe2l2 5′-GCATGATGGACTTGGAGTT-3′

### TMT quantitative proteomic analysis

IFE cells were cultured in a medium containing 100 μg/ml holotransferrin (experimental group) or without holotransferrin (control group) for 2 days. Three samples from each group were used for TMT quantitative proteomic analysis. The TMT quantitative proteomic analysis was performed by Shanghai Applied Protein Technology Co., Ltd. (project number: P20200801875).

### Cell viability assay

CCK8 (Dojindo, Japan, 1/10) was used to assess the viability of treated IFE cells. IFE cells were seeded in 96-well plates and treated and cultured for 48 h. After removing the medium, 100 μL of fresh medium and 10 μL of CCK8 were added to each cell, and cell viability was measured after incubation at 37 °C for 1 h. A Spark microplate reader (Tecan, Austria) was used to measure cell viability at an absorbance of 450 nm.

### Assessment of cell proliferation

IFE cells were inoculated in 96-well plates at 4 × 10^5^ cells per well, cultured in a medium with one of several concentrations of holotransferrin (0 µg/ml, 1 µg/ml, 10 µg/ml, 100 µg/ml, or 1 mg/ml) for 48 h and observed. Images were acquired using the Incubation Cell S3 Live Cell Analysis System (Sartorius AG, Göttingen, Germany). The area of confluence was calculated, normalized to 0 h, and displayed as a ratio to calculate the proliferation rate.

### Western blot analysis

Protein expression levels were determined by western blot analysis. To measure relative protein expression levels, treated IFE cells were fully lysed in RIPA buffer (Thermo Fisher Scientific) to obtain protein lysates. Protease inhibitors and phosphatase inhibitors (1:100) were added during protein extraction (MedChemExpress), and a Pierce BCA Protein Analysis Kit (Thermo Fisher Scientific) was used to measure protein concentrations. Protein samples were separated by 10% SDS–PAGE and transferred to PVDF membranes. The membranes were blocked in 5% skim milk and incubated with the respective primary antibodies overnight at 4 °C. The samples were incubated with horseradish peroxidase-conjugated secondary antibodies (1:5000 dilution; Cell Signaling Technology) for 1 h at room temperature, and an iBright FL1500 imaging system (Invitrogen) and Super Signal West Femto Maximum Sensitivity Substrate (Thermo Fisher Scientific, Invitrogen) were used to detect and analyze protein expression levels. Antibody information was provided for anti-ALDH3B1 (Proteintech Group; 19,446–1-AP), anti-NRF2 (Cell Signaling Technology; 12721S), anti-KRT10 (Abcam, Cambridge, UK; ab76318, WB: 1/1000), anti-KRT15 (Abcam; ab52816, IF/FC: 1/200), rabbit anti-P63 (Abcam; ab124762, WB: 1/1000), rabbit anti-KRT14 (Abcam; ab181595, WB: 1/1000), anti-ferritin heavy chain (anti-FTH; Carlsbad, CA, USA, WB: 1/1000), rabbit anti-ferritin light chain (anti-FTL; MA5-32,755, WB: 1/1000), and rabbit anti-GAPDH (Cell Signaling Technology; WB: 1/1000).

### Flow cytometry measurement of intracellular lipid peroxidation levels

IFE cells were treated and cultured for 48 h, digested with trypsin (Gibco, Canada), collected in 2-ml sample tubes, and centrifuged (1500 × g, 5 min). BODIPY 581/591 C11 (Invitrogen, USA, 1:1000) and FerroOrange (Dojindo, Japan, 1:500) probes were used to detect intracellular lipid peroxidation levels and divalent iron ion levels, respectively, in IFE cells. The cells were first stained with BODIPY 581/591 C11 or FerroOrange probe and then with eBioscience Flow Cytometry Staining Buffer (with the precipitate being resuspended in 300 µl of the buffer after centrifugation), incubated at 37 °C for 40 min, and detected with a CytoFLEX flow cytometer (Beckman Coulter, Indianapolis, CA). First, we distinguished dead cells, cell debris, and live cells by adjusting the voltage so that the dead cells and cell debris would accumulate at the lower left position of the FSC-SSC voltage gate, and then the live cells were examined, followed by the values within the FITC gate and PE gate. Then, cells treated with lipid peroxidation probes C11-BODIPY and FerroOrange were examined, followed by the values within the FITC gate and PE gate. Oxidation of the polyunsaturated butadiene fraction of C11-BODIPY resulted in a shift of the fluorescence emission peak from ~ 590 nm to ~ 510 nm, as detected via the PE and FITC channels. Excitation light at 543 nm and emission light at 580 nm for FerroOrange was detected via the PE channel. The relative levels of lipid peroxidation and divalent ferric ions were quantified by calculating the arithmetic mean of the FITC/PE and PE values, respectively, across three experiments.

### Immunofluorescence

Cells were fixed in 4% paraformaldehyde for 15 min at room temperature, washed 3 times in PBS, permeabilized in 0.5% Triton X-100 and PBS for 10 min, and then blocked with BlockAid Blocking Solution (Invitrogen) for 1 h. The cells were incubated with primary antibodies overnight at 4 °C and then with the secondary antibody anti-rabbit Alexa Flour-488 (diluted 1/200, Cell Signaling Technology) at room temperature for 1 h. Finally, nuclei were stained with Hoechst (Invitrogen, Thermo Fisher Scientific) diluted 1:10,000 at room temperature for 30 min. Immunofluorescence images were taken with an A1R confocal microscope (Nikon, Melville, NY).

#### PCR

Total RNA was extracted using RNAiso Plus (9109, Takara, Japan) according to the manufacturer’s protocol. Sketch™ RT Master Mix (RR036A, Takara, Japan) was used to synthesize complementary DNA by reverse transcription of RNA into DNA. Quantitative real-time PCR (qRT–PCR) experiments were performed using TB-Green™ Premix™ II (RR820A, Takara, Japan). Primers were designed and synthesized by Takara. Actin was used as an endogenous reference gene. Relative gene expression was determined using the 2^−ΔΔCT^ method. The sequences of the employed PCR primers are shown below.

Actin-F 5′-AAATGGTGAGGGTCGGTGAAC-3′

Actin-R 5′-CAAATCCTCTTTGCCACTG-3′

ALDH3B1-F 5′- GAACTACCCCGTGAACCTGAC -3′

ALDH3B1-R 5′- ACCTTCTCCGTGCCCTTACTA -3′

NRF2-F 5′-TAGATGACCATGAGTCGCTTGC -3′

NRF2-R 5′-GCCAAACTTGCTCCATGTCC -3′

### Inhibitors of the NRF2 pathway

Cells were inoculated in six-well plates, and after 24 h of cell culture, an inhibitor of NRF2 (Hinokitiol, MedChemExpress; 20 µM, 12 h) was added to the cells.

### Statistical analysis

All data were statistically analyzed using GraphPad Prism version 9.0.0 (GraphPad Software, San Diego, CA). Data were obtained from three experiments and are expressed as the mean ± standard deviation, and between- and among-group comparisons were performed using unpaired *t* tests and one-way ANOVA, respectively. Asterisks indicate significant differences between conditions in each group. *P* values are marked as *P < 0.05, **P < 0.01, ***P < 0.001.

## Results

### Identification of IFE cells

**Fig. 1:**
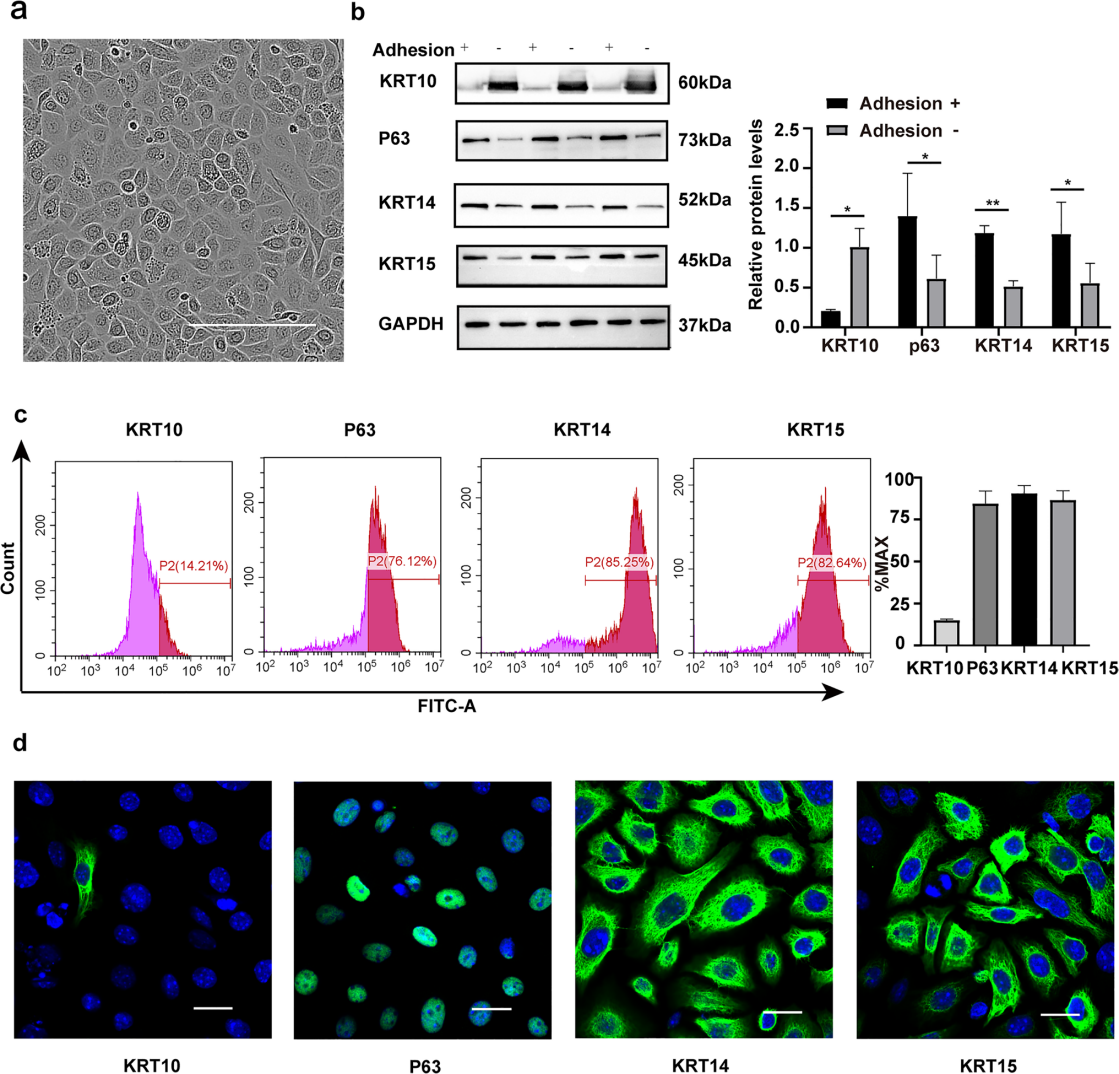
Identification of IFE cells. **a** IFE cells were cultured for 2 days. Scale bar = 50 μm. **b** Western blotting was performed to determine the expression of KRT14, KRT15, P63, and KRT10 in adherent and nonadherent cells. **c** Flow cytometry was performed to identify the percentages of KRT14-positive, KRT15-positive, P63-positive, and KRT10-positive primary cells. **d** Immunofluorescence identification of KRT14, KRT15, P63, and KRT10 expression in extracted cells is shown. Scale bar = 20 μm. All experiments were performed in triplicate. The data are presented as means ± SDs, and the significance of differences was evaluated using unpaired *t* tests. *P 0.05, **P < 0.01

We extracted IFE cells from the skin of newborn mice. As shown in Fig. 1a, after 2 days of culture, IFE cells appeared as pebble-like cells under light microscopy. KRT14, KRT15, and P63 are epidermal stem cell markers, and KRT10 is a keratinocyte marker (Wang et al. 2022). We extracted protein lysates from rapidly adherent and nonadherent cells. KRT14, KRT15, and P63 were highly expressed in rapidly adherent cells, while the expression of KRT10 was low in these cells (Fig. 1b). In addition, flow cytometry showed that more than 85% of primary cells expressed KRT15, KRT14, and P63, while less than 15% expressed KRT10 (Fig. 1c). We extracted primary IFE cells from neonatal mouse skin for study and performed immunofluorescence staining using the basal cell markers KRT14, KRT15, and P63 and the mature keratinocyte marker KRT10. The results showed that most of the primary cells were KRT14 positive, KRT15 positive, or P63 positive, while few keratin-forming cells were KRT10 positive (Fig. 1d). These experimental results demonstrate that the purity of the IFE cells we extracted from the skin of newborn mice was high.

### Effects of different concentrations of holotransferrin treatment on the survival rate and lipid peroxidation level of IFE cells

**Fig. 2:**
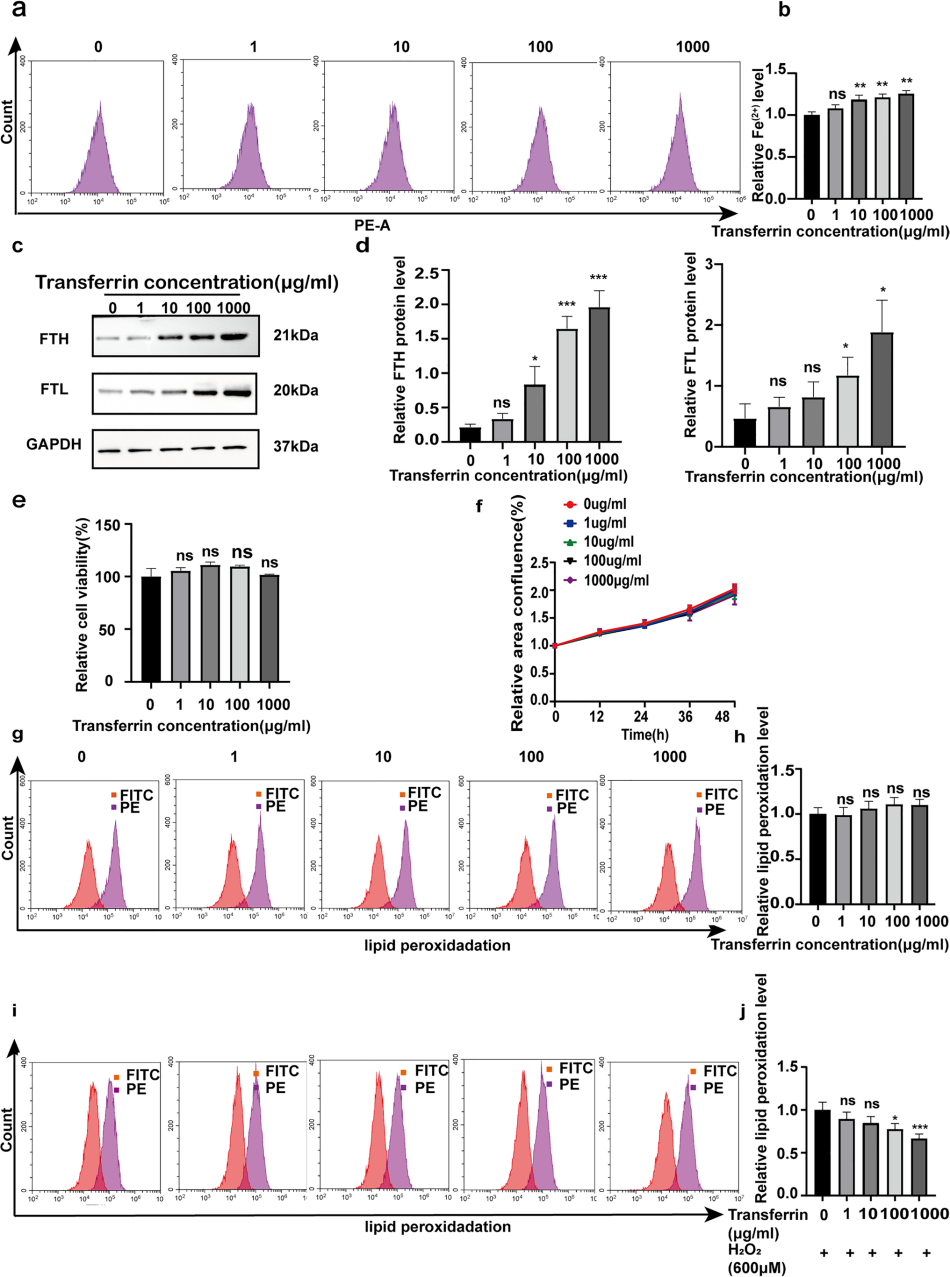
The survival rate, proliferation rate, and intracellular lipid peroxidation level of IFE cells were affected by treatment with different concentrations of full-valent ferritin for 48 h. **a**, **b** IFE cells were treated with 0, 1, 10, 100, or 1000 μg/ml holotransferrin, and changes in intracellular divalent iron ion content were detected by flow cytometry through the PE channel. **c**, **d** Protein blotting assays were conducted to detect changes in FTH and FTL expression in IFE cells after treatment with different concentrations of holotransferrin. **e** The survival rate of IFE cells after holotransferrin treatment was measured by CCK8. **f** Cell proliferation ability was measured by confluence measurement normalized to 0 h and calculated using IncuCyte. **g**, **h** IFE cells were treated with 0, 1, 10, 100, or 1000 µg/ml holotransferrin and assayed by flow cytometry through the FITC and PE channels, with the ratio of FITC to PE representing the intracellular lipid peroxidation level. **i**, **j** IFE cells were treated with 600 μm of H_2_O_2_ and holotransferrin for 6 h, and intracellular lipid peroxidation levels were measured by flow cytometry. All experiments were performed in triplicate. The data are presented as means ± SDs, and the significance of differences was evaluated using unpaired *t* tests. *P < 0.05, **P < 0.01, and ***P < 0.005; ns, not significant

IFE cell viability and lipid peroxidation levels do not change with increasing iron ion concentration in IFE cells. Flow cytometry was used to detect the content of divalent iron (Fe^2+^) in IFE cells treated with different concentrations of holotransferrin (0 µg/ml, 1 µg/ml, 10 µg/ml, 100 µg/ml, and 1 mg/ml). As shown in Fig. 2a, b, as the holotransferrin concentration increased, the content of intracellular divalent iron also increased. We detected protein expression by immunoblotting in each group of IFE cells. We also examined the expression of FTH and FTL in IFE cells by protein immunoblotting and found that their expression gradually increased with increasing holotransferrin concentration (Fig. 2c, d). In addition, after we treated IFE cells with different concentrations of holotransferrin, we assayed IFE cell viability with CCK8 and found no difference among the groups (Fig. 2e). Under these conditions, we also investigated IFE cell proliferation and found almost no difference in proliferation rate among the groups (Fig. 2f). Moreover, we examined the level of lipid peroxidation in IFE cells and found almost no difference among the groups (Fig. 2g, h). These experimental results showed that although the intracellular iron content increased, the level of lipid peroxidation, cell viability, and proliferation of IFE cells was not affected. A protective mechanism of IFE cells against lipid peroxidation may exist. To investigate whether the elevated iron ion concentration had any protective effect on IFE cells or whether some protective mechanism was activated in IFE cells that were stimulated as described above, IFE cells were treated with different concentrations of holotransferrin along with 600 µM H_2_O_2_ for 6 h. The results showed a trend of decreasing intracellular lipid peroxidation level with increasing holotransferrin concentration (Fig. 2i, j). The experimental results suggest that a protective mechanism against lipid peroxidation damage exists in IFE cells and that the higher the iron content in IFE cells is, the stronger its protective effect is.

### Elevated expression of ALDH3B1 in IFE cells and ALDH3B1-overexpressing cells

**Fig. 3:**
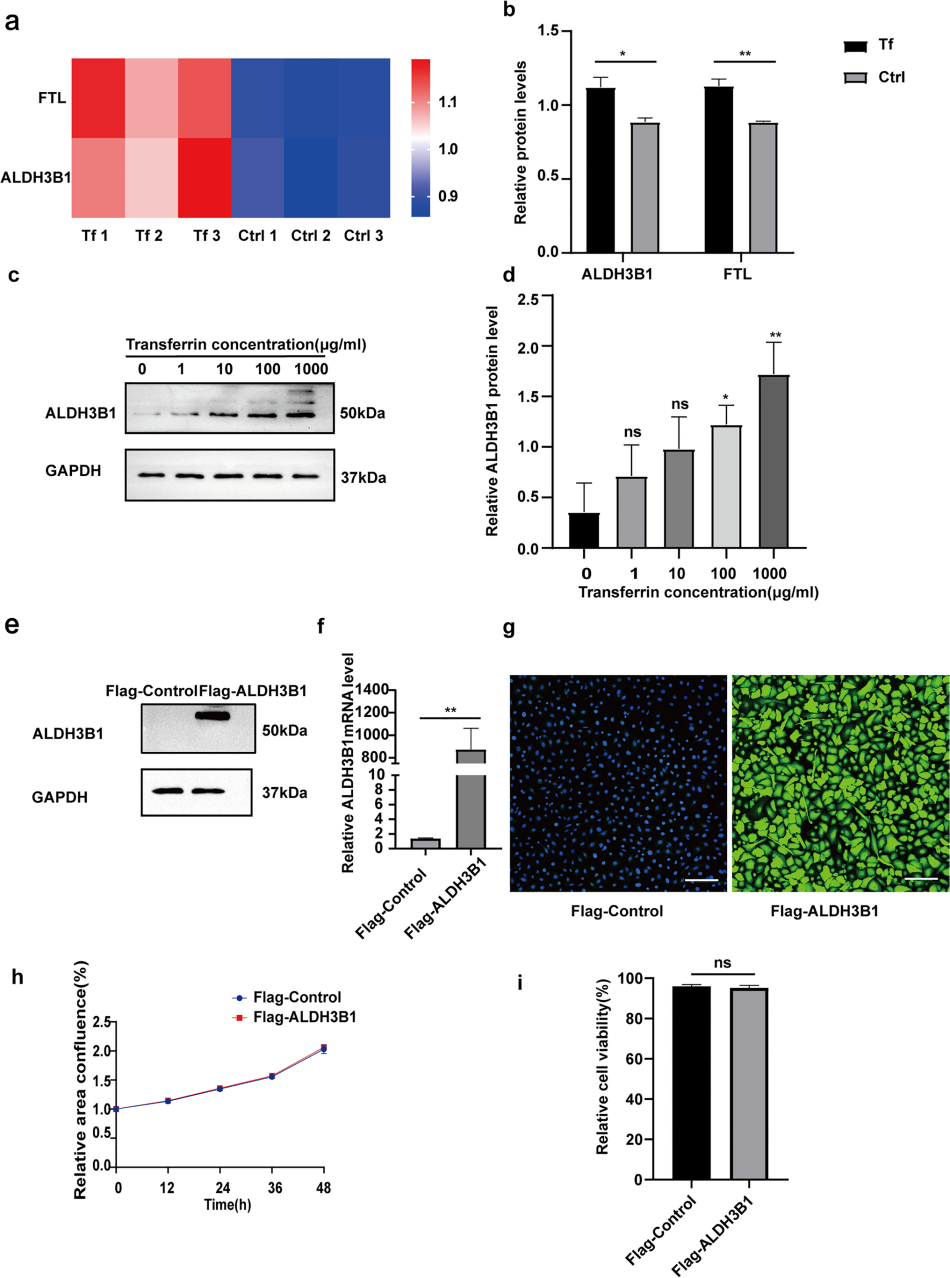
Elevated expression of ALDH3B1 after holotransferrin treatment of IFE cells and ALDH3B1 overexpression. **a, b** IFE cells were cultured with a medium containing 100 μg/ml holotransferrin (Tf group) or normal medium (Ctrl group) for 48 h, and TMT quantitative proteomic analysis showed that the expression of ALDH3B1 and FTL was elevated. **c**, **d** Western blotting assay was conducted to detect the expression of ALDH3B1 at different concentrations of holotransferrin. **f** qPCR analysis of ALDHEB1 mRNA levels is shown. **e**, **g** Western blotting and immunofluorescence results showing the expression of Flag in the experimental and control groups are shown. Scale bar = 100 μm. **h** Cell proliferation ability was measured by confluence measurement normalized to 0 h and calculated using IncuCyte. **i** Cell viability was determined by CCK8 assay and compared between the Flag-control group and the Flag-ALDH3B1 group. All experiments were performed in triplicate. The data are presented as means ± SDs, and the significance of differences was evaluated using unpaired *t* tests. *P < 0.05, **P < 0.01, and ***P < 0.005; ns, not significant

Based on the results of the previous experiment, we conducted additional experiments. IFE cells were cultured with a medium containing 100 μg/ml holotransferrin (Tf group) or with a normal (Ctrl group) medium for 48 h. The TMT quantitative proteomics results showed that the expression of ALDH3B1 and FTL was elevated in the Tf group compared with the Ctrl group (Fig. 3a, b). In addition, we cultured IFE cells with different holotransferrin concentrations (0 µg/ml, 1 µg/ml, 10 µg/ml, 100 µg/ml, and 1 mg/ml) for 48 h, and after protein extraction, protein blotting analysis confirmed that ALDH3B1 expression showed an elevated trend (Fig. 3c, d). The elevated expression of FTL further demonstrates that the addition of holotransferrin to cultured IFE cells increases the intracellular concentration of iron ions. As the concentration of holotransferrin increased, the expression of ALDH3B1 gradually increased. This finding demonstrates that ALDH3B1 may be a key gene in the resistance of IFE cells to lipid peroxidation. We next explored whether ALDH3B1 has a role in the resistance of IFE cells to lipid peroxidation. First, we infected IFE cells with Flag-ALDH3B1 adenovirus or Flag-control adenovirus. Western blot and qPCR analyses showed that ALDH3B1 protein expression and mRNA transcription in the Flag-ALDH3B1 group were significantly higher than those in the Flag-Control group (Fig. 3e, f). The immunofluorescence results revealed Flag expression in both the Flag-ALDH3B1 and Flag-Control groups, and the fluorescence of the Flag-ALDH3B1 group was more intense (Fig. 3g). Moreover, the results of the cell proliferation and cell viability experiment showed that there was no difference in the cell proliferation curve between the two groups (Fig. 3h, i).

### Protective effect of ALDH3B1 on IFE cells against lipid peroxidation

**Fig. 4:**
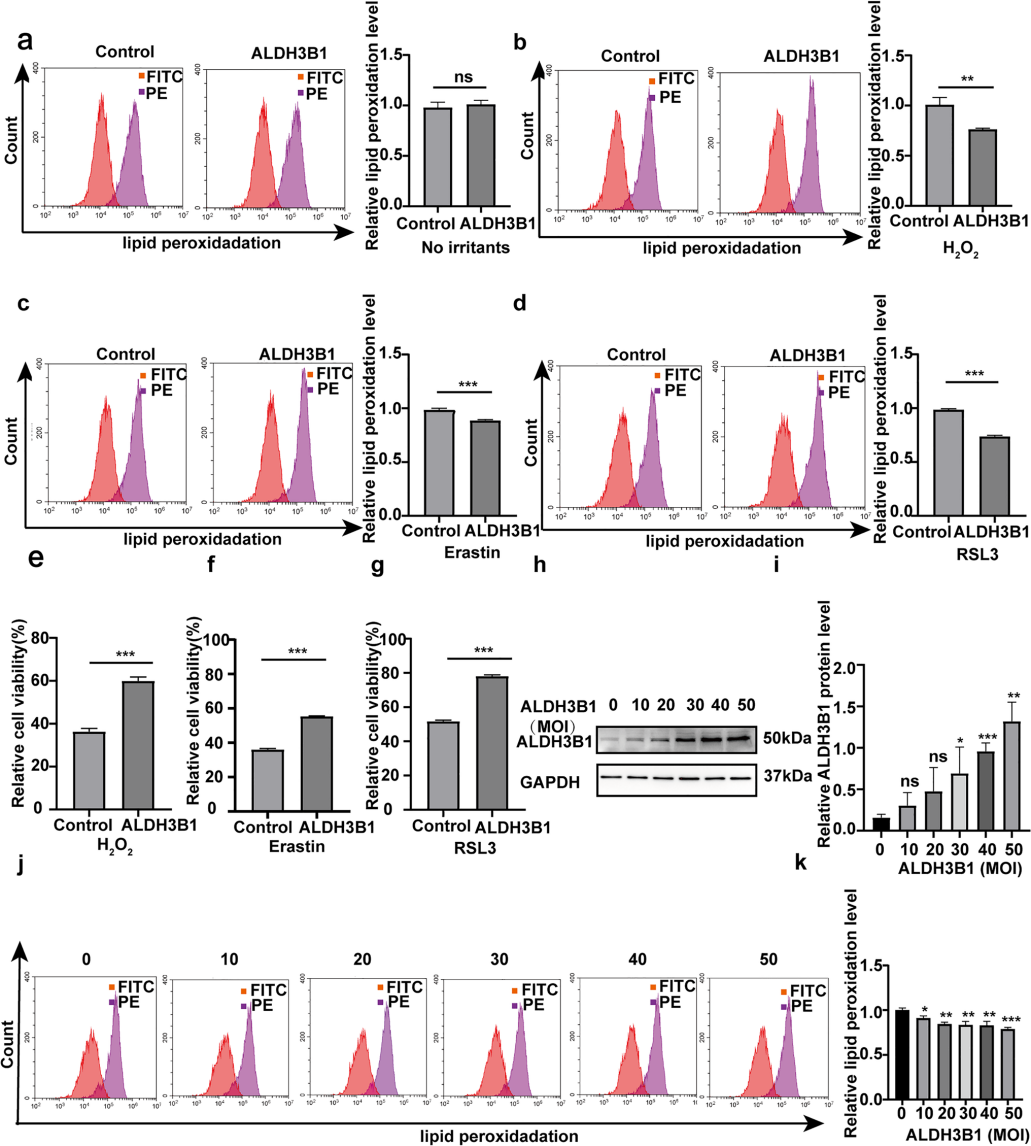
Protective effect of ALDH3B1 on IFE cells against lipid peroxidation. **a** Flow cytometry through the FITC and PE channels was used to detect lipid peroxidation levels in the ALDH3B1 group and the control group under conditions without any stimulation. **b**–**d** After 6 h of Rsl3 (5 µM), erastin (20 µM), and H_2_O_2_ (600 µM) treatment, lipid peroxidation levels were measured by flow cytometry through the FITC and PE channels and compared between the groups. **e**–**g** Cell viability under Rsl3, erastin, and H_2_O_2_ treatment conditions was measured and compared between the groups. **h**, **i** IFE cell transfection was conducted at an MOI value of 0 to 50, and a protein immunoblotting assay was used to detect the expression of ALDH3B1. **j**, **k** Cells were transfected at different MOI values under 600 μM H_2_O_2_ treatment conditions, and the lipid peroxidation levels of each group were detected via fluorescence in the FITC and PE channels. All experiments were performed in triplicate. The data are presented as means ± SDs, and the significance of differences was evaluated using unpaired *t* tests. *P < 0.05, **P < 0.01, and ***P < 0.005; ns, not significant

To verify the protective effect of ALDH3B1 on IFE cells, we divided the cells into ALDH3B1 and control groups. As shown in Fig. 4a, we first verified that overexpression of ALDH3B1 had no effect on lipid peroxidation levels in IFE cells under unstimulated conditions. After treatment with H_2_O_2_, RSL3, and erastin, IFE cells in the ALDH3B1 group showed lower levels of lipid peroxidation than those in the control group (Fig. 4b–d). This result demonstrates that overexpression of ALDH3B1 enables IFE cells to resist lipid peroxidation when factors that increase lipid peroxidation are present. Furthermore, the CCK8 results showed that IFE cells in the ALDH3B1 group had higher cell viability than those in the control group (Fig. 4e–g). We then verified that ALDH3B1 expression is related to the ability of IFE cells to resist lipid peroxidation. We used different MOI values for cell transfection and found that as the expression of ALDH3B1 gradually increased (Fig. 4h, i), the ability of IFE cells to resist lipid peroxidation gradually increased, and the level of lipid peroxidation was gradually reduced by 600 μM H_2_O_2_ treatment (Fig. 4j, k). The above experiments demonstrated that overexpression of ALDH3B1 in IFE cells can contribute to the resistance to lipid peroxidation.

### Activation of ALDH3B1 via the NRF2 pathway protects IFE cells

**Fig. 5:**
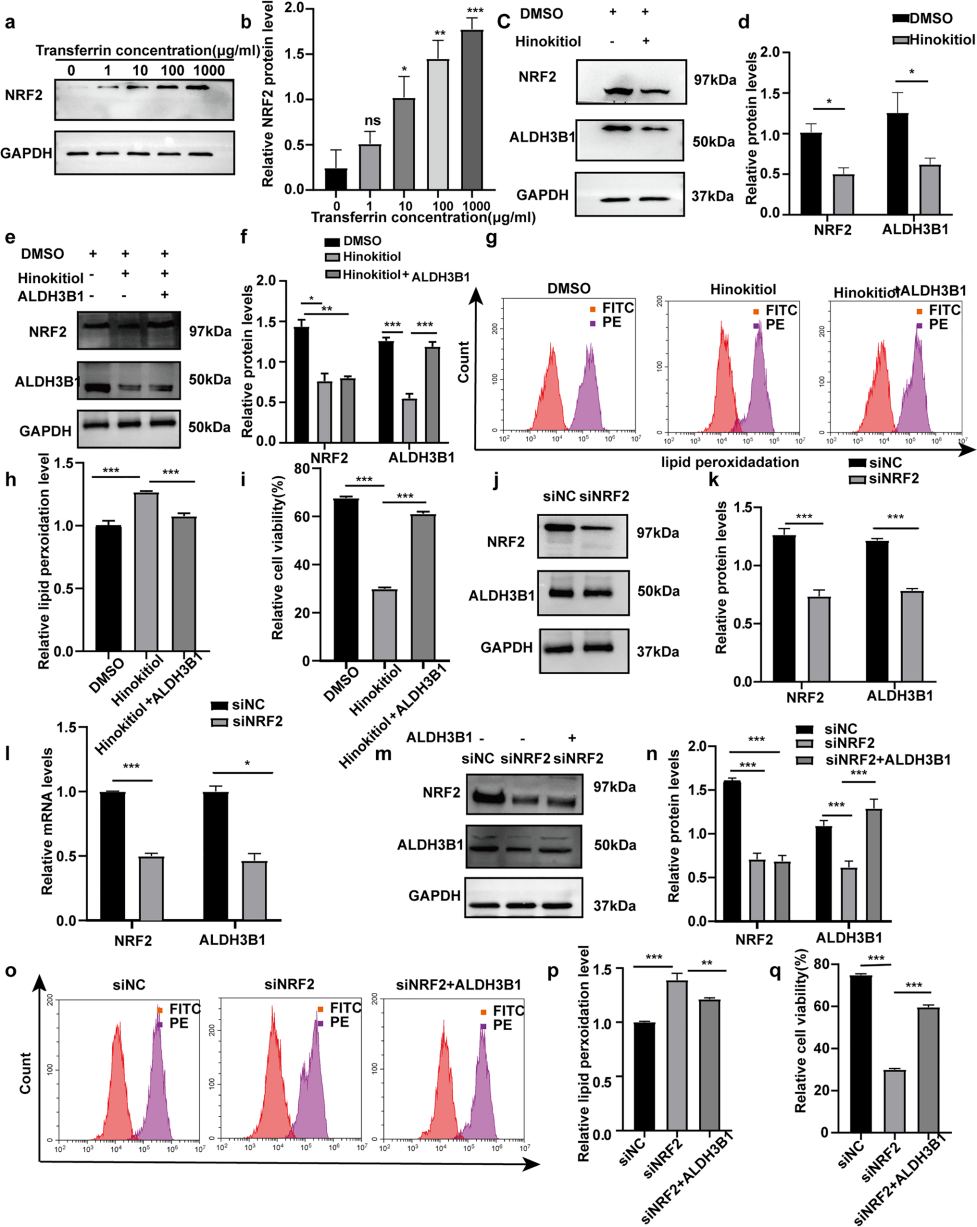
Elevation of ALDH3B1 expression through activation of the NRF2 signalling pathway protects IFE cells against damage from lipid peroxidation. **a**, **b** Expression of NRF2 in IFE cells treated with 0, 1, 10, 100, or 1000 μg/ml holotransferrin is shown. **c**, **d** Western blotting analysis of NRF2 and ALDH3B1 expression in the hinokitiol and DMSO groups is shown. **e**, **f** Western blotting analysis of NRF2 and ALDH3B1 expression in the hinokitiol, DMSO, and ALDH3B1 + hinokitiol groups is shown. **g**, **h** The levels of lipid peroxidation in the hinokitiol, DMSO, and ALDH3B1 + hinokitiol groups were detected by flow cytometry. **i** Cell viability in the hinokitiol, DMSO, and ALDH3B1 + hinokitiol groups was detected by CCK8. **j**, **k** Western blotting was conducted to analyze NRF2 and ALDH3B1 expression in the siNC and siNRF2 groups. **l** qPCR was performed to analyze NRF2 and ALDH3B1 mRNA levels. The data were normalized to the amount of β-actin mRNA. **m**, **n** Western blotting analysis of NRF2 and ALDH3B1 expression in the siNC, siNRF2, and ALDH3B1 + siNRF2 groups is shown. **o**, **p** The levels of lipid peroxidation in the siNC, siNRF2, and ALDH3B1 + siNRF2 groups were detected by flow cytometry. **q** Cell viability in the siNC, siNRF2, and ALDH3B1 + siNRF2 groups was detected by CCK8. All experiments were performed in triplicate. The data are presented as means ± SDs, and the significance of differences was evaluated using unpaired *t* tests. *P < 0.05, **P < 0.01, and ***P < 0.005

The NRF2 pathway is an important pathway for cellular antioxidation (Tumbar et al. 2004). After treatment of IFE cells with different concentrations of holotransferrin, we found that while the expression of ALDH3B1 gradually increased, the expression of NRF2 also gradually increased (Fig. 5a, b). We speculated that ALDH3B1 may be linked to the NRF2 pathway.

To test the above speculation, we first verified that under the treatment conditions of 1 mg/ml holotransferrin, IFE cells treated with an inhibitor of NRF2 (hinokitiol group) expressed NRF2 and showed decreased expression of ALDH3B1 compared to cells treated with DMSO (DMSO group) (Fig. 5c, d). After we inhibited the expression of NRF2 in IFE cells with hinokitiol, the expression of ALDH3B1 was reduced in the hinokitiol group, while the lipid peroxidation level of cells was elevated and cell viability was reduced in this group. Subsequently, by treating cells in the ALDH3B1 overexpression group with hinokitiol, an inhibitor of NRF2, in Western blotting experiments, we found that the expression of ALDH3B1 was higher in the DMSO and ALDH3B1 + hinokitiol groups than in the hinokitiol group (Fig. 5e, f). In addition, we examined the lipid peroxidation levels and viability of cells in the DMSO, hinokitiol, and hinokitiol + ALDH3B1 groups by flow cytometry and CCK8, respectively. The DMSO and hinokitiol + ALDH3B1 groups had lower lipid peroxidation levels and higher cell survival than the hinokitiol group (Fig. 5g–i). Through this experiment with hinokitiol inhibitor, we provided the first demonstration that ALDH3B1 protects IFE cells from damage by lipid peroxidation through NRF2 pathway activation and that ALDH3B1 plays a role in the protection of IFE cells against lipid peroxidation via NRF2.

To verify our findings, NRF2 activity in IFE cells was silenced using siNRF2 to create a siNRF2 group, and a siNC group served as a negative control group. First, we investigated the silencing efficiency of siNRF2 by Western blotting and qPCR experiments. (Fig. 5j–l). Western blotting experiments were performed to examine the expression of NRF2 and ALDH3B1 in the siNC group, siNRF2 group, and siNRF2 + ALDH3B1 group. NRF2 expression was found to be higher in the siNC group than in the siNRF2 group and siNRF2 + ALDH3B1 group, while ALDH3B1 expression was lower in the siNRF2 group than in the siNC and siNRF2 + ALDH3B1 groups (Fig. 5m, n). We also examined the lipid peroxidation levels and cell viability in the siNC group, siNRF2 group, and siNRF2 + ALDH3B1 group by flow cytometry and CCK8 assay. The siNC group and siNRF2 + ALDH3B1 group had lower levels of lipid peroxidation and higher cell survival than the siNRF2 group (Fig. 5o–q). By setting up the siNC and siNRF2 groups, we demonstrated that ALDH3B1 protects IFE cells from damage by lipid peroxidation through NRF2 pathway activation. Based on this finding, we established a siNRF2 + ALDH3B1 group, and the experimental results demonstrated that ALDH3B1 plays a role in NRF2-mediated protection of IFE cells against lipid peroxidation.

## Discussion

IFE cells are located in the basal layer of the epidermis and proliferate and differentiate during migration to the suprabasal layer, where they promote wound healing (Ma et al. 2019; Ito et al. 2005; Mascré et al. 2012; Taylor et al. 2000). Stem cells in hair follicles and IFE cells contribute to the re-epithelialization of wounds (Bagdas et al. 2015). IFE cells mainly consist of stem cells and progenitor cells, which are the sources of almost all differentiated keratinized cells in the epidermis (Page et al. 2013; Tumbar et al. 2004). During wound healing, cells from hair follicles and interfollicular epidermis have been shown to migrate to the wound surface, and damage to IFE cells can lead to nonhealing of the wound surface (Wlaschek et al. 2019; Chen et al. 2016; Liu et al. 2021). Therefore, IFE cells play an important role in the wound-healing process.

The balance between the positive and harmful effects of ROS is crucial for wound healing. Although the production of ROS is important for initiating wound healing, excessive ROS can impair the wound healing process by causing lipid peroxidation in cells, which in turn affects protein modification and leads to DNA damage, ultimately increasing cell death and senescence (Chen et al. 2016). Excessive and uncontrolled lipid peroxidation during wound healing maintains the wound in an inflammatory phase, leading to delayed healing and the formation of chronic wounds (Cano Sanchez et al. 2018; Bagdas et al. 2015; Moseley et al. 2004). Additionally, trace elements and transition metal iron play important roles in cell proliferation and differentiation, but when iron metabolism is abnormal, iron can disrupt redox homeostasis through the Fenton reaction, leading to lipid peroxidation and chronic trauma formation. The rupture of blood cells in chronic wounds and the persistent inflammatory effects lead to high levels of iron ions in the wound and iron loading by macrophages (via erythrophagocytosis), resulting in unrestricted macrophage promotion of inflammatory activation. The generated ROS cause a cascade of deleterious reactions that increase lipid peroxidation, resulting in high levels of lipid peroxidation in chronic wounds and nonhealing wounds (Wright et al. 2014; Wlaschek et al. 2019; Ding et al. 2021). Some harmful factors that expose IFE cells to lipid peroxidation can lead to delayed healing or nonhealing of wounds. Therefore, how to maintain the balance of pro-oxidant and antioxidant capacity and increase the ability of trabecular cells to resist lipid peroxidation during wound healing has become an important issue.

ALDH3B1 is a key enzyme for cellular resistance to aldehydes and oxidative inducers (Marchitti et al. 2010; Sun et al. 2020). In this study, we found that IFE cell activity and proliferative capacity were not affected when the cellular concentration of iron ions was elevated. In addition, under high iron ion concentrations, the expression of ALDH3B1 in IFE cells was elevated. Therefore, we hypothesized that ALDH3B1 helps to protect IFE cells from lipid peroxidation damage. Treatment of IFE cells with holotransferrin causes elevated intracellular levels of divalent iron, and holotransferrin catalyzes lipid peroxidation in the presence of divalent iron or esteroxygenase, a highly expressed unsaturated fatty acid on the cell membrane, thereby inducing cellular iron death (Wright et al. 2014; Aroun et al. 2012). We selected the iron death inducers Rsl3 and erastin to culture IFE cells. We observed that the ALDH3B1 group had higher cell viability and lower levels of lipid peroxidation than the control group. In addition, the NRF2 pathway has been reported to play a key role in the body’s resistance to lipid peroxidation, and this pathway can reduce oxidative damage and is involved in the regulation of antioxidant gene expression during oxidative stress, thereby enhancing cellular resistance to lipid peroxidation (Jazvinšćak Jembrek et al. 2021; Maresova et al. 2020; Li et al. 2019; Xu et al. 2021). We, therefore, hypothesized that activation of the NRF2 pathway elevates the expression of ALDH3B1 and thus promotes the resistance of IFE cells to lipid peroxidation damage. When IFE cells were treated with different concentrations of holotransferrin, an increase in ALDH3B1 expression was detected along with an increase in NRF2 expression as holotransferrin concentration increased. Therefore, we selected a holotransferrin concentration of 1 mg/ml and an NRF2 inhibitor for further investigation. We found a decrease in NRF2 expression accompanied by decreases in ALDH3B1 expression, lipid peroxidation levels, and intracellular ROS levels in the experimental group compared to the control group. This result confirms that it is through the activation of the NRF2 pathway that ALDH3B1 expression is increased and exerts its effects against lipid peroxidation in IFE cells. This study demonstrates that ALDH3B1 acts through the NRF2 pathway to resist lipid peroxidation in IFE cells and thus provides a possible target for the treatment of lipid peroxidation in chronic wounds.

This study has some drawbacks; although we showed the resistance conferred by ALDH3B1 to lipid peroxidation in IFE cells, we did not include any animal experiments to confirm the role of ALDH3B1 in wound healing. In addition, although we demonstrated that NRF2 can affect ALDH3B1 expression, the mechanism underlying its effect on ALDH3B1 was not identified.

## Conclusion

In this study, we established a cellular lipid peroxidation injury cell model using H_2_O_2_, RSL3, and erastin to investigate the resistance to lipid peroxidation conferred by ALDH3B1. Our results show that ALDH3B1 can protect IFE cells from lipid peroxidation damage and that the NRF2 pathway plays a role in promoting ALDH3B1 expression to improve IFE cell survival and reduce lipid peroxidation levels.
